# Quantification of Trastuzumab–HER2 Engagement In Vitro and In Vivo

**DOI:** 10.3390/molecules25245976

**Published:** 2020-12-17

**Authors:** Alena Rudkouskaya, Jason T. Smith, Xavier Intes, Margarida Barroso

**Affiliations:** 1 Department of Molecular and Cellular Physiology, Albany Medical College, Albany, NY 12208, USA; rudkoua@amc.edu; 2 Center for Modeling, Simulation, and Imaging in Medicine, Rensselaer Polytechnic Institute, Troy, NY 12180, USA; smithj28@rpi.edu (J.T.S.); intesx@rpi.edu (X.I.)

**Keywords:** trastuzumab, HER2, fluorescence lifetime, FRET imaging, target engagement, immunoconjugate

## Abstract

Human EGF Receptor 2 (HER2) is an important oncogene driving aggressive metastatic growth in up to 20% of breast cancer tumors. At the same time, it presents a target for passive immunotherapy such as trastuzumab (TZM). Although TZM has been widely used clinically since 1998, not all eligible patients benefit from this therapy due to primary and acquired drug resistance as well as potentially lack of drug exposure. Hence, it is critical to directly quantify TZM–HER2 binding dynamics, also known as cellular target engagement, in undisturbed tumor environments in live, intact tumor xenograft models. Herein, we report the direct measurement of TZM–HER2 binding in HER2-positive human breast cancer cells and tumor xenografts using fluorescence lifetime Forster Resonance Energy Transfer (FLI-FRET) via near-infrared (NIR) microscopy (FLIM-FRET) as well as macroscopy (MFLI-FRET) approaches. By sensing the reduction of fluorescence lifetime of donor-labeled TZM in the presence of acceptor-labeled TZM, we successfully quantified the fraction of HER2-bound and internalized TZM immunoconjugate both in cell culture and tumor xenografts in live animals. Ex vivo immunohistological analysis of tumors confirmed the binding and internalization of TZM–HER2 complex in breast cancer cells. Thus, FLI-FRET imaging presents a powerful analytical tool to monitor and quantify cellular target engagement and subsequent intracellular drug delivery in live HER2-positive tumor xenografts.

## 1. Introduction

Human epidermal growth factor receptor 2 (HER2) is a member of receptor tyrosine kinase family comprised also of EGFR (HER1), HER3, and HER4. For the last three decades, HER2 has been studied in great detail by clinicians and biologists, as HER2 gene amplification and protein overexpression have been associated with aggressive metastatic breast and ovarian cancers, as well as gastric and bladder cancers [1]. Targeting HER2 with various therapeutic antibodies, e.g., trastuzumab (TZM) and pertuzumab, as well as antibody–drug conjugates (T-DM1) has led to a dramatic improvement in survival outcomes for HER+ cancer patients [2,3,4]. Nevertheless, a significant number of eligible patients do not benefit from this therapy due to primary or acquired drug resistance. Despite extensive research, the molecular mechanisms of trastuzumab action and resistance are still not completely understood [5,6,7]. One of the possible reasons is insufficient target exposure and, as a result, reduced target engagement and poor drug efficacy. Therefore, the ability to directly quantify antibody-HER2 engagement in the tumors may provide significant insights into whether drug therapy resistance is due to insufficient drug delivery in tumors.

Successful intracellular drug delivery is paramount for an increased efficacy of anti-cancer therapy [8]. The molecular imaging of immunoconjugates in preclinical studies has been crucial in this regard [9]. Traditionally, nuclear imaging dominates the field both for clinical and preclinical studies [10,11]. Lately, optical molecular imaging has made impressive progress in the development of novel drug delivery imaging assays [12]. However, there is still a pressing need to develop new imaging methodologies to monitor the dynamic behavior of drug–receptor engagement and receptor dimerization during cancer progression and in response to treatment. Fluorescence lifetime imaging (FLI) offers a unique tool to non-invasively sense drug–receptor engagement via Förster Resonance Energy Transfer (FRET) [13]. As a nanometer-range proximity assay, FRET can examine protein–protein interactions in vitro and in vivo. Upon donor excitation, fluorescence lifetime (FLI) senses FRET occurrence by the reduction of donor fluorophore lifetime when in the proximity of 2–10 nm of an acceptor molecule [13,14]. In case of drug–receptor engagement imaging assays, FLI quantifies FRET occurrence by estimating the reduction of the fluorescence lifetime of the donor fluorophore only when donor-labeled and acceptor-labeled ligands bind to dimerized receptors and are thus brought into close proximity to allow energy transfer to occur, including intra- as well as inter-receptor FRET [15,16,17,18,19]. In contrast to fluorescence intensity-based microscopy, optical imaging, or PET-based measurements of target engagement, which require multi-compartmental pharmacokinetics models [20], FLI FRET imaging discriminates between passive accumulation and receptor-mediated uptake, providing a direct readout of receptor engagement by assessing the fraction of labeled-donor entity undergoing binding to its respective receptor [15,16,17,18,19].

FLI-FRET in living systems has been extensively validated by our group over the last few years using transferrin–transferrin receptor (Tf-TfR) system in cancer cells, tumor xenografts, and other organs [18,19,21,22]. Recently, we have reported that in vivo near-infrared (NIR) macroscopy FLI (MFLI) imaging quantitatively reports on NIR-labeled ligand–receptor interaction via FRET [13,18,19]. Furthermore, the FLI-based optical approach is amenable to imaging multiple biomarkers simultaneously, paving the way to not only quantifying drug target engagement but also drug response [23]. Recently, we reported on a new protocol to simultaneously image target engagement and glucose metabolism in an undisturbed tumor environment, which allows for the in vivo monitoring of ligand intracellular tumor uptake in combination with changes in tumor metabolic status in real time [24].

Herein, to the best of our knowledge, we report for the first time on the quantification of the binding of HER2 to TZM to monitor HER2–TZM engagement via FLI-FRET microscopy (FLIM-FRET) as well as MFLI-FRET imaging in HER2-overexpressing AU565 breast cancer cells, both in 2D cell culture and live, intact preclinical models, respectively.

## 2. Results

### 2.1. TZM–AF700 Binding and Trafficking in AU565 Breast Cancer Cells

**Figure 1:**
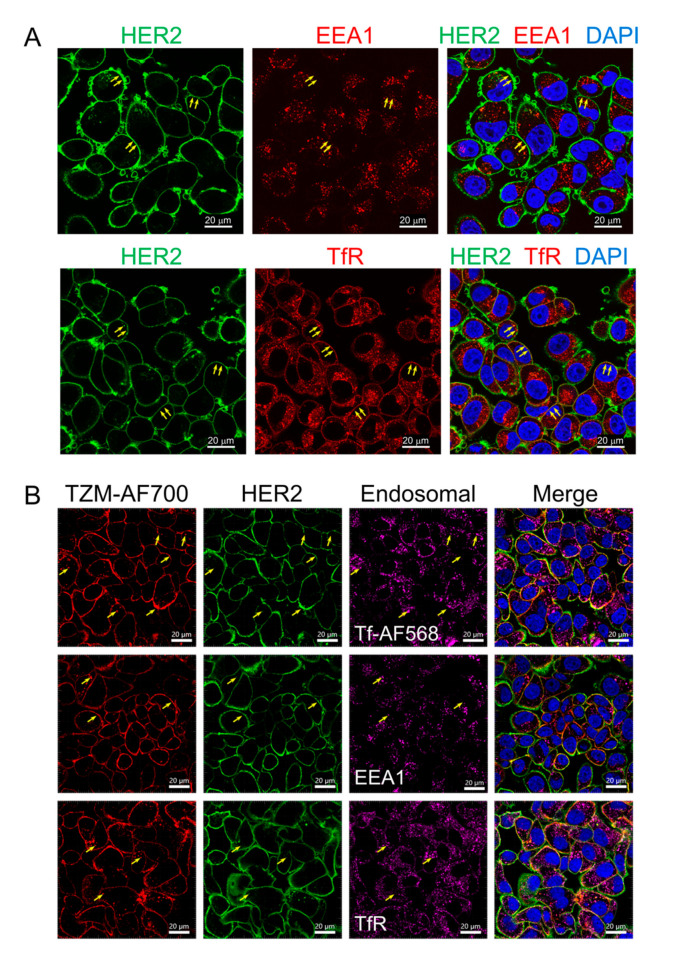
Confocal microscopy of Human EGF Receptor 2 (HER2) and trastuzumab (TZM)–AF700 distribution in AU565 cells. (**A**) Immunofluorescence analysis of HER2 (green; arrows), early endosomal antigen 1 (EEA1), or transferrin receptor (TfR) (red; arrows). Middle slices of z-stacks consisting of 6–8 optical slices are shown. (**B**) AU565 cells were loaded with 5 µg/mL TZM–AF700 (red) and 5 µg/mL Tf–AF568 (magenta) for 24 h and then processed for immunostaining of HER2 (green) and endosomal markers EEA1 or TfR (magenta). Maximum intensity projections of z-stacks consisting of 6–8 optical slices are shown. Nuclei are visualized with DAPI. Scale bar = 20 µm.

**Figure 2:**
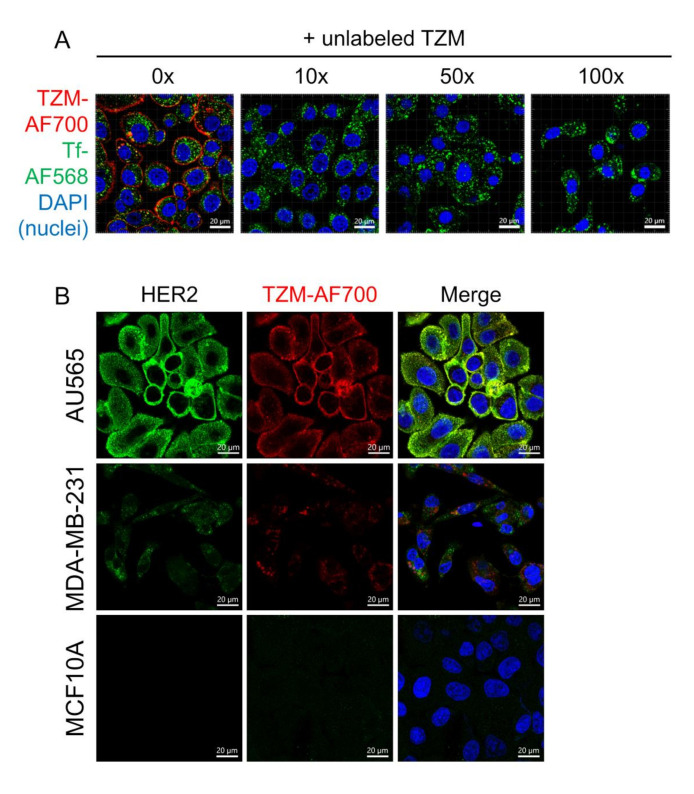
Comparison of intracellular uptake of TZM–AF700 into HER2-positive and HER2-negative cell lines. (**A**) To test the specificity of HER2-mediated TZM–AF700 binding and internalization, competition experiments were performed. AU565 cells were loaded with 5 µg/mL TZM–AF700 (red) and 5 µg/mL Tf-AF568 (green) in the presence of increasing amounts of unlabeled TZM. Single slice confocal microscopy displays TZM–AF700 (red) and Tf–AF568 (green) binding and internalization into AU565 cells in the presence of increasing 0×, 10×, 50×, and 100× unlabeled TZM. (**B**) HER2-positive AU565 and HER2-negative MDA-MB-231 and MCF10A cells were subjected to 24 h uptake of 20 µg/mL TZM–AF700 (red) and then processed for anti-HER2 immunostaining (green). Nuclei were visualized with DAPI (blue). Maximum intensity projections of z-stacks consisting of 6–8 optical slices are shown. Confocal images were collected using identical settings on an LSM880 confocal microscope. Scale bar = 20 µm.

To characterize TZM–HER2 engagement and trafficking, we performed extensive microscopy studies in AU565 cell monolayers (Figure 1 and Figure 2) using several endosomal markers, such as early endosomal antigen 1 (EEA1), a well-known marker of the early endosomal pathway, and transferrin (Tf) and its receptor (TfR), which are well-characterized markers of the early/recycling endocytic pathway [25,26]. TfR localizes mainly to the plasma membrane and early/recycling endocytic pathway, including the early endosomes as well as fast and slow recycling vesicles. TfR–Tf complexes undergo clathrin-mediated endocytosis and then are recycled back to the cell surface [27,28].

In Figure 1A, we show that in the absence of TZM, HER2 does not appear to localize with early endocytic marker EEA1, colocalizing predominantly with TfR at the plasma membrane (yellow arrows), as demonstrated by Pearson correlation coefficient measurements (Supplementary Figure S1A). In Figure 1B, these endosomal markers were used to characterize the pattern of internalization and trafficking of TZM–AF700 conjugates. Tf–AF568, a fluorescently tagged Tf ligand, was co-internalized with TZM–AF700 for 24 h into AU565 cells, followed by immunofluorescence with anti-HER2, anti-TfR, and anti-EEA1. The main goal of this experiment was to directly visualize and compare the intracellular distribution of TZM–AF700 on the cell surface and endosomal pathway. Most TZM–AF700 conjugates are detected at the cell surface, displaying strong colocalization with HER2 via Pearson coefficient measurements (Supplementary Figure S1B). Furthermore, a few punctate structures containing TZM–AF700 and HER2 can be detected inside the AU565 cells. These vesicles may represent endosomal compartments due to colocalization with Tf-AF568, which shows significant but reduced Pearson coefficient measurements when compared with that of TZM vs. HER2 colocalization (Figure 1B, yellow arrows; Supplementary Figure S1B). 

To demonstrate that TZM–AF700 is binding to HER2 at the cell surface, competition assays in the presence of increasing concentrations of unlabeled TZM were performed in AU565 cells (Figure 2). As shown in Figure 2A, TZM–AF700 binding to AU565 cells is mediated by HER2 as indicated by the reduced cellular association of TZM–AF700 in the presence of increasing levels of unlabeled TZM. Interestingly, increasing amounts of unlabeled TZM did not affect Tf–TfR endocytosis and trafficking, although they clearly led to a dramatic reduction of cell surface binding and internalization of TZM–AF700 into AU565 cells (Figure 2A). These results are consistent with previous studies establishing that HER2 is rapidly recycled back to the surface upon internalization [29,30]. 

To directly address the specificity of the TZM–AF700 NIR labeled probe, TZM–AF700 uptake assays were performed in AU565, MDA-MB-231, and MCF10A cells. As shown by immunoblotting analysis, HER2 is overexpressed in breast cancer AU565 cells but not in aggressive triple negative breast cancer MDA-MB-231 cells or in non-cancerous mammary epithelial MCF10A cells (Supplementary Figure S2A). However, TZM–AF700 and anti-HER2 probes can detect a reduced but detectable signal in MDA-MB-231 but not in MCF10A cells using LSM880 confocal microscopy (Figure 2B). Importantly, anti-HER2 and TZM–AF700 distribution patterns strongly colocalize in AU565 but not in MDA-MB-231 cells, as shown via Pearson correlation coefficient analysis (Supplementary Figure S2B). Thus, these results suggest that MDA-MB-231 cells show a non-specific but still measurable fluorescence signal upon the binding of TZM–AF700 or anti-HER2 probes in cell uptake and immunofluorescence-based assays, respectively. The detection of non-specific HER2 probe binding to MDA-MB-231 cells is probably due to the high sensitivity of novel GaAsP detectors used in advanced confocal microscopes and the presence of putative HER2-independent binding targets at cell surface.

### 2.2. Quantification of TZM–HER2 Engagement in Cell-Based Assays

**Figure 3:**
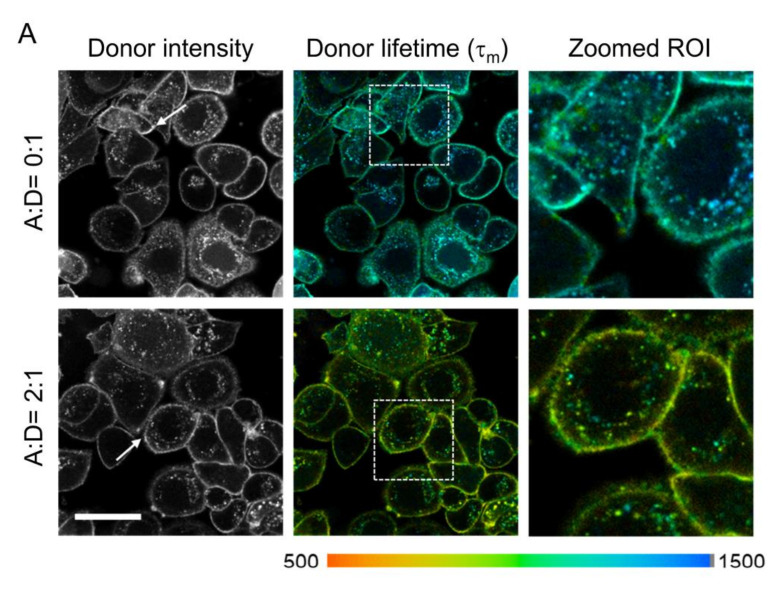

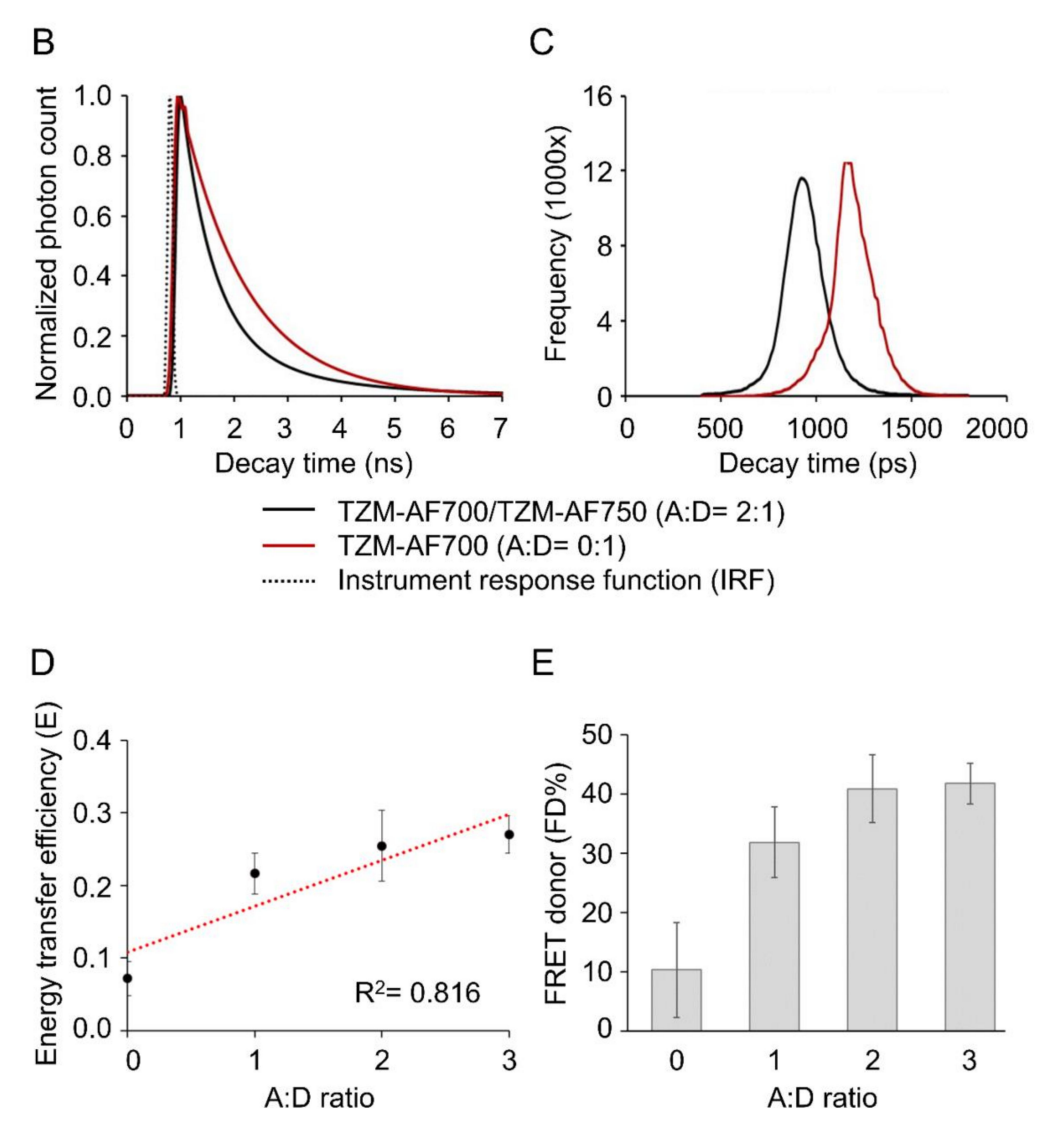
TZM Forster Resonance Energy Transfer (FLI-FRET) microscopy (FLIM-FRET) analysis in AU565 cancer cells. (**A**) The representative time-correlated single photon counting (TCSPC) images of fluorescence intensity and mean lifetime map (τ_m_) in cells treated with TZM-AF700 only [Acceptor/Donor (A:D) = 0:1; donor single-labeled] or with TZM–AF700 plus TZM–AF750 (A:D = 2:1; double-labeled); pseudo-color range = 500–1500 ps. Zoomed regions of interest (ROIs) of both single and double-labeled cells show heterogeneity of fluorescence lifetime of TZM–AF700 within the cells. White arrows indicate x, y coordinates used for the curve fitting using SPCImage. Scale bar = 50 µm. (**B**) Representative fitting curves and IRF, the fluorescent lifetime decay in the single and double-labeled cells was determined by comparing the fitting of the decay data using both single- and double-exponential decay models, respectively. (**C**) Fluorescent lifetime distribution in AU565 cells treated with TZM–AF700 (A:D = 0:1) or TZM–AF700 and TZM–AF750 (A:D = 2:1). (**D**) Quantification of FRET donor (FD%) levels in AU565 cells treated with a near-infrared (NIR) TZM–FRET pair at various A:D ratios. Analysis was performed using 10 distinct pixel coordinates (*n* = 10) from five independent ROIs; error bars represent confidence interval at 95%. (**E**) Quantification of TZM–FRET efficiency (E) in relation to A:D ratios. Data presented as mean ± confidence interval at 95%, *n* = 10.

FRET, as a nanometer range proximity assay, is an ideal tool to measure probe–receptor engagement and track their fate upon internalization in cancer cells. Here, we used NIR FLIM-FRET imaging to investigate TZM–HER2 binding and uptake in an HER2-overexpressing AU565 human breast cancer cell culture model. To probe TZM–AF700–HER2 engagement, a 24 h uptake assay of TZM–AF750 (acceptor) and TZM–AF700 (donor) at various Acceptor/Donor (A:D) ratios with the donor concentration constant at 20 µg/mL was performed (Figure 3).

**Figure 4:**
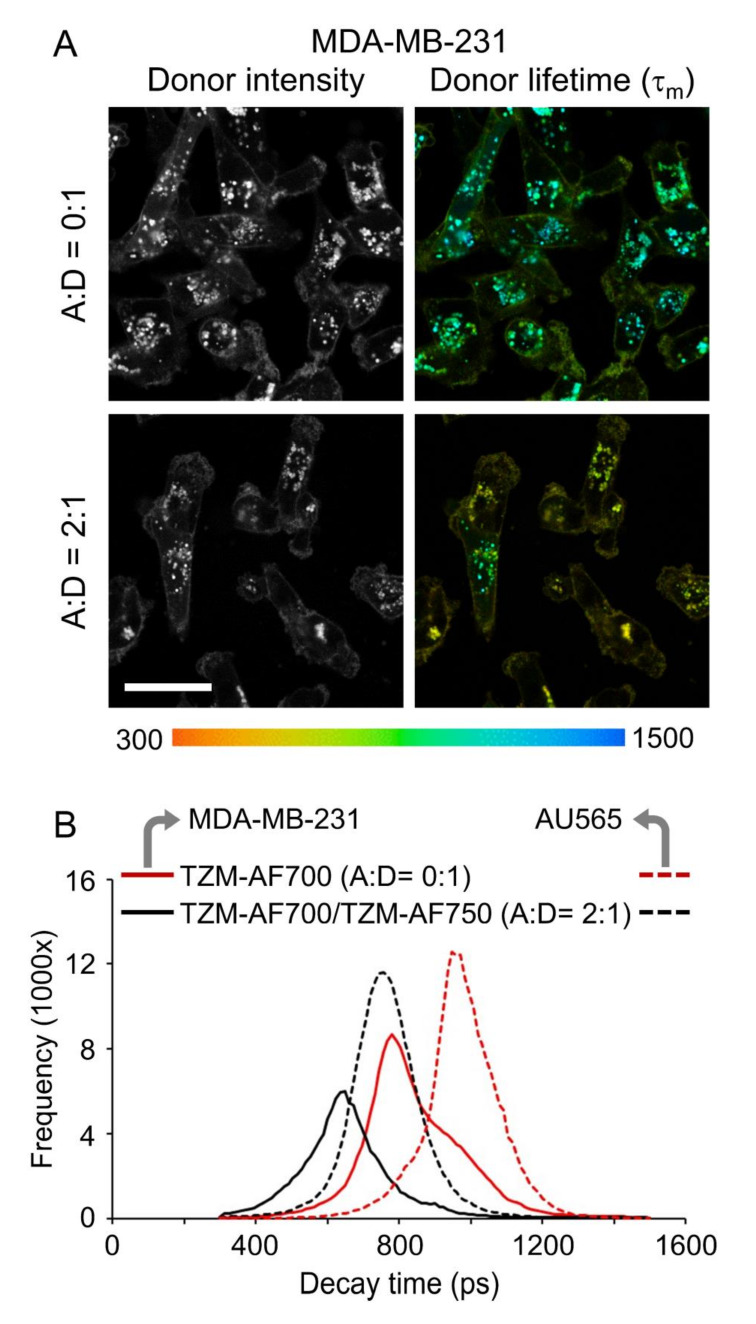
TZM FLIM-FRET analysis in MDA-MB-231 cancer cells. (**A**) The representative TCSPC images of fluorescence intensity and mean lifetime map (τm) in cells treated with TZM–AF700 (A:D = 0:1) or with TZM–AF700 plus TZM–AF750 (A:D = 2:1) pseudo-color range = 300–1500 ps. Scale bar = 50 µm. (**B**) Comparison of fluorescent lifetime distribution in MDA-MB-231 (solid lines) and AU565 cells (dashed lines) treated with TZM–AF700 (A:D = 0:1 red), TZM–AF700 plus TZM–AF750 (A:D = 2:1 black).

Representative time-correlated single photon counting (TCSPC) images of fluorescence lifetime maps (Figure 3A) show a reduction of donor mean lifetime (τ_m_) in the presence of acceptor (A:D = 2:1) indicating FRET occurrence. TZM–AF700 and TZM–AF750 are brought together into the FRET range (2–10 nm) upon binding to dimerized HER2. This results in substantial lifetime reduction, which suggests an increased fraction of interacting NIR-labeled TZM in comparison to reduced levels of the non-interacting TZM population. Importantly, the FRET signal due to inter-receptor FRET cannot be excluded. Notably, the shortest lifetimes were found at the cell surface, indicating that FRET events due to TZM–HER2 binding occur mainly at the plasma membrane. Surprisingly, the same trend was also observed in donor only AF700-labeled samples (A:D = 0:1), indicating that the binding of TZM to the receptor may result in conformational changes leading to a partial reduction of donor τ_m_ (mean fluorescence lifetime) in the absence of acceptor. Moreover, the significant heterogeneity of TZM–AF700 τ_m_ in endosomes across the cells was noted, which likely reflects changes in the endosomal microenvironment and/or partial dissociation of TZM–HER2 complexes during trafficking. The fluorescent lifetime decay graph (Figure 3B) and frequency graph (Figure 3C) demonstrate a significant reduction of donor lifetime in the presence of acceptor (A:D = 2:1), indicating FRET events due to HER2–TZM binding. As expected for FRET measurements of receptor–ligand interactions, e.g., TfR–Tf complexes [17,19,24], both HER2–TZM FRET donor fraction (FD%) and FRET efficiency (*E*) display rising trendlines when plotted against increasing A:D ratios (Figure 3D,E). Interestingly, the average FD% values are similar for both TfR–Tf and HER2–TZM complexes in cancer cells (Supplementary Figure S3) [17,19,24]. To exclude molecular crowding effects, we have previously demonstrated that FLIM-FRET behaves independently from increasing acceptor concentration [16,31]. Then, to compare the FLIM-FRET signal across HER2 negative cells, both MDA-MB-231 and MCF10A cells were subjected to a similar FLIM-FRET imaging assay, as described in Figure 3A. MCF10A showed no detectable fluorescence signal upon incubation with TZM–AF700 (Figure 2B) and thus was used as a clear negative control for FLIM-FRET analysis. Indeed, MCF10A cells displayed a very reduced photon count level, which was below the threshold necessary for adequate FLIM fitting analysis (residual signal collected in MCF10A cells is shown in Supplementary Figure S4). In contrast, donor fluorescence lifetime was already significantly quenched in MDA-MB-231 cells incubated only with TZM–AF700 donor (Figure 4). Moreover, the FLIM signal in MDA-MB-231 cells is predominantly detected in intracellular structures in contrast to AU565 cells, suggesting a different, non-specific TZM-AF700 uptake pathway. Taken together, these results show that FRET can detect TZM–HER2 binding at the plasma membrane of AU565 cells using NIR FLIM-FRET imaging.

### 2.3. Quantification of TZM–HER2 Engagement in AU565 Breast Cancer Xenografts

**Figure 5:**
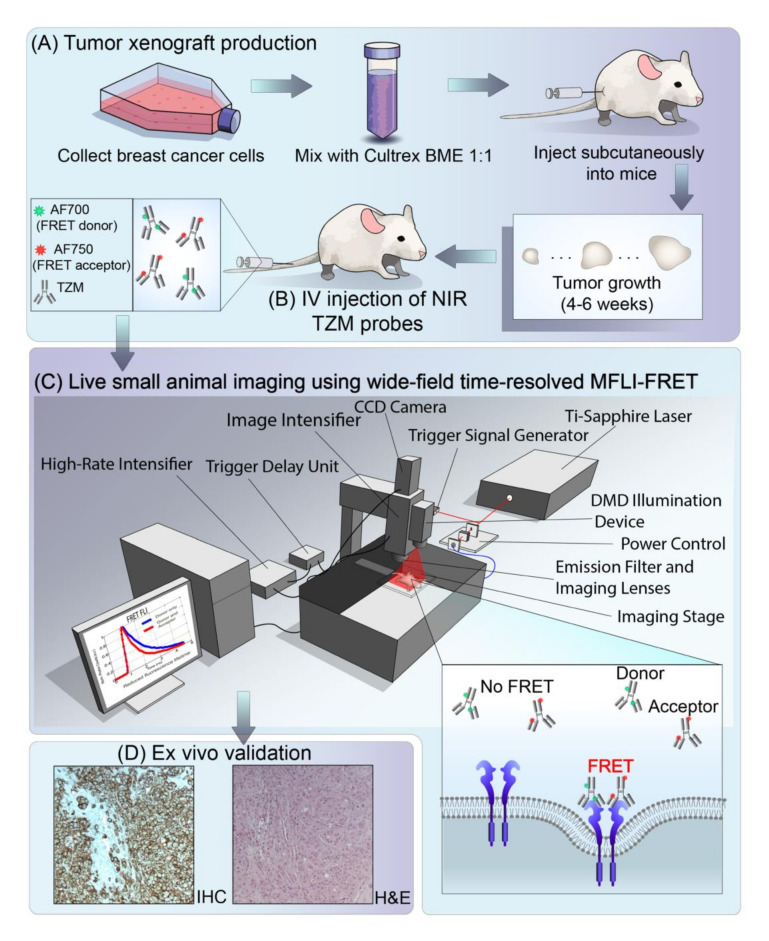
Schematic representation of live small animal NIR wide-field time-resolved macroscopic fluorescence lifetime FRET imaging (MFLI-FRET) for preclinical studies. (**A**) AU565 tumor xenograft production; (**B**) Intravenous tail-vein injection of AF700–TZM and/or AF750–TZM; (**C**) Live small animal imaging using wide-field time-resolved FLI-FRET macroscopy (MFLI-FRET) imager; (**D**) Ex vivo validation using immunohistochemical (IHC) and H&E staining. Inset top left: illustration of TZM–AF700 and TZM–AF750 ligands. Inset bottom right: illustration of TZM–HER2 FLI-FRET events upon binding of donor- and acceptor-labeled antibodies to HER2 dimer at the surface of cancer cells. DMD: digital micromirror device; CCD: charge-coupled device.

After validation of TZM–HER2 FLIM-FRET in vitro, we tested whether TZM–HER2 binding could be detected in vivo using MFLI-FRET imaging of nude mice bearing AU565 tumor xenografts. Figure 5 illustrates the principles of detecting molecular events of antibody–receptor binding in undisturbed tumor xenografts using MFLI-FRET imager. Firstly, tumor xenografts are implanted in nude mice using AU565 HER2 positive human breast cancer cells (Figure 5A). Secondly, upon adequate tumor growth, mice are intravenously injected with TZM–AF700 (donor only; A:D = 0:1) or TZM–AF700/TZM–AF750 donor/acceptor FRET pairs at A:D = 2:1 (Figure 5B). Thirdly, 24–72 h post-injection, anesthetized animals are subjected to whole-body non-invasive MFLI-FRET imaging (Figure 5C). Finally, tumors are extracted and subjected to ex vivo validation using immunohistochemical (IHC) analysis (Figure 5D).

**Figure 6:**
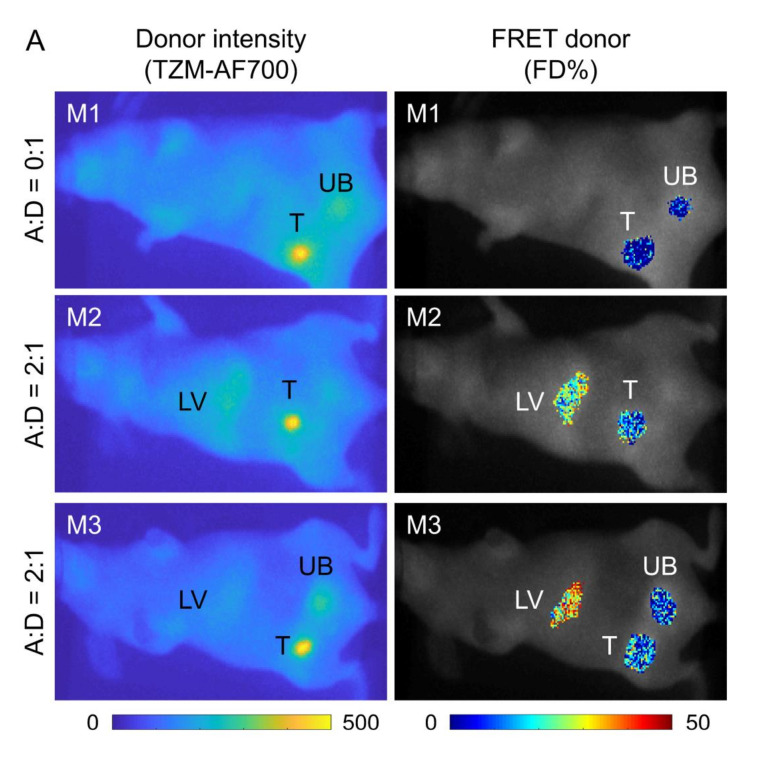

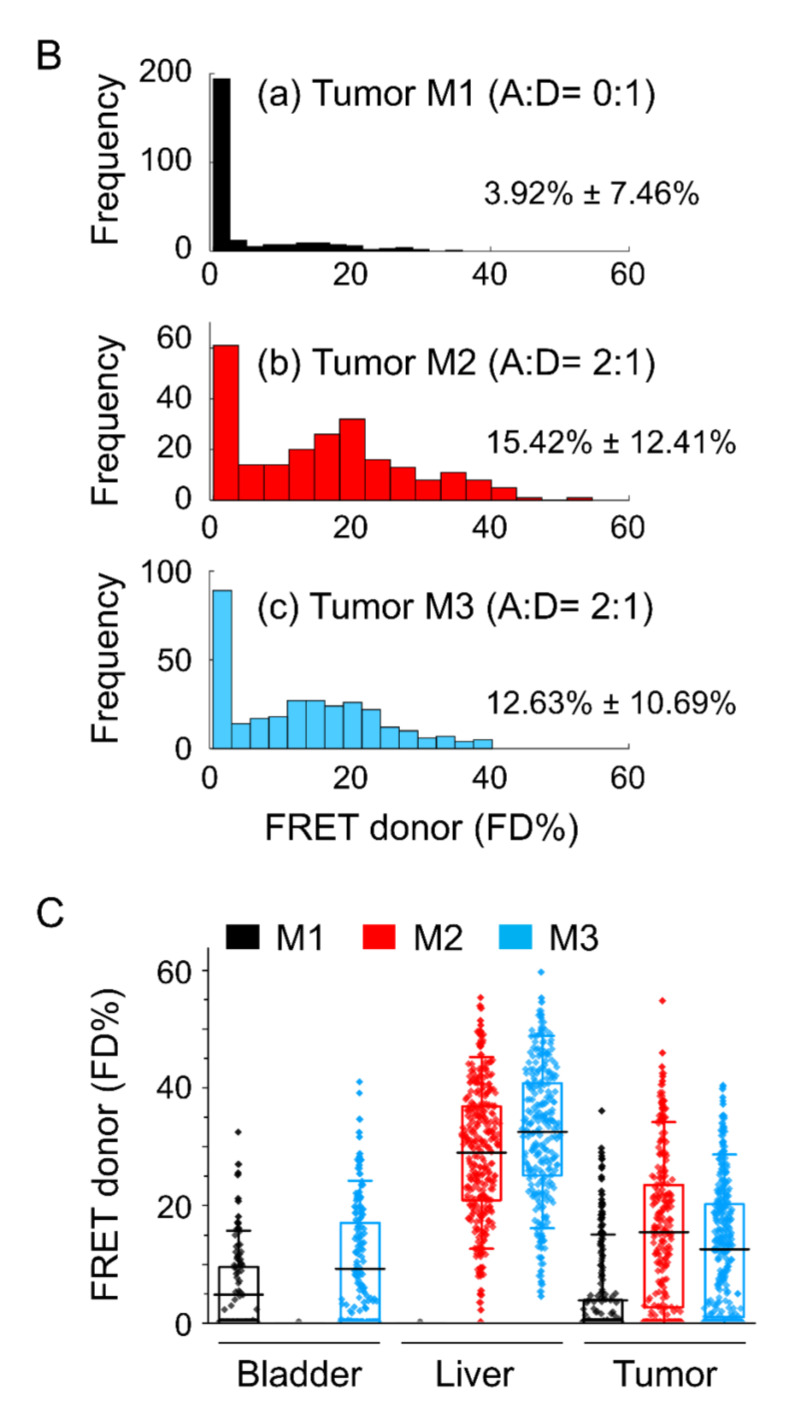
Whole body quantification of TZM–HER2 engagement via MFLI-FRET in vivo imaging. (**A**) Mice were injected with 20 µg TZM–AF700 alone (M1) or TZM–AF700 and 40 µg TZM–AF750 and subjected to MFLI-FRET imaging at 48 h p.i. Panels show TZM donor maximum intensity ROIs (both soluble and bound probe) and FRET donor fraction (FD%) map (bound and internalized probe) in the tumors (T), livers (LV), and urinary bladders (UB). (**B**) Histograms of FD% retrieved for each tumor. Numbers represent mean ±SD. (**C**) Quantification of FD% in the tumors, bladders and livers. Data presented as box indicating 25–75% pixel values, horizontal and vertical lines indicate mean with ±1.5 SD, respectively.

To test TZM–HER2 binding in vivo using MFLI-FRET imaging, three mice bearing AU565 tumor xenografts were intravenously injected with 20 µg TZM–AF700 alone (donor only; A:D = 0:1; M1 mouse) or TZM–AF750/TZM–AF700 at A:D ratio 2:1 (M2 and M3 mice) and imaged at 48 h post-injection (Figure 6A). In striking difference to NIR Tf MFLI FRET imaging, we observed excellent signal-to-noise ratio, practically with zero background (Figure 6A).

Unlike TfR, HER2 is not ubiquitously expressed in all tissues, so the fluorescence signal is restricted to the tumor composed of HER2 overexpressing human breast cancer cells, and occasionally, to some extent, to livers and bladders due to degradation and excretion. Importantly, substantial FRET levels as a measure of TZM-HER2 binding were found in the tumors of mice injected with TZM–FRET pair (M2 and M3), but not in the negative control mouse M1 injected with donor only (Figure 4B,C). This confirms specificity of NIR TZM MFLI-FRET imaging. Interestingly, we also found significant FRET levels in the livers of M2 and M3, but not M1, and in the bladder of M3. The reason for that is unclear, and we plan to explore it further in future TZM–MFLI imaging experiments. Nevertheless, these results show for the first time, the ability to detect TZM–HER2 binding in vivo using MFLI-FRET imaging of live intact AU565 tumor xenografts.

### 2.4. Histological Evaluation of AU565 Breast Cancer Xenografts

**Figure 7:**
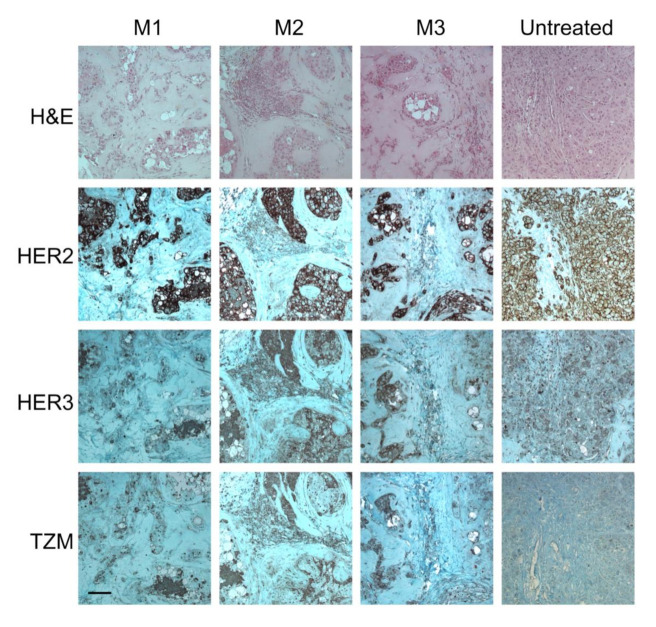
IHC ex vivo validation confirms TZM accumulation in the cancer cells both positive for HER2 and HER3. Consecutive sections of tumors M1–M3 and tumor from untreated mouse were processed for H&E staining, anti-HER2, anti-HER3, and anti-TZM immunohistochemical staining. NovaRED was used as peroxidase substrate (brown stain), tissue was counterstained with methyl green. Scale bar = 100 µm.

To validate the MFLI FRET TZM imaging experiment, we performed extensive IHC analysis of excised tumors (Figure 7). Since the mice were injected with TZM probes twice with an interval of one month, tumors tissue showed massive cell death. However, the remaining cancer cells still displayed a notable expression of HER2 and HER3 compare to untreated tumors. Significantly, among surviving tumor cells, TZM staining appears to associate predominantly with a population of tumor cells expressing both HER2 and HER3. These results are consistent with the possibility that TZM treatment may select for HER2 and HER3 co-expression and/or a more aggressive HER2–HER3 heterodimer phenotype, which is a hypothesis that we will be exploring in the future using MFLI-FRET in vivo imaging.

## 3. Discussion

Trastuzumab is one of the most successful anti-HER2 cancer therapeutics used for the last three decades, especially in conjunction with complimentary pertuzumab treatment. Yet, only a fraction of cancer patients eligible for anti-HER2 therapy are responsive to the treatment, even in combination with pertuzumab [32]. With high degree of intra- and inter-tumor heterogeneity of HER2 expression, dimerization, and trafficking, many strategies are needed to tackle these issues. In tumors with high density of HER2 at the cell surface, the depletion of sortilin-related receptor 1 (SORLA) appears to direct HER2 to the degradative endolysosomal pathway and sensitizes cancer cells to lysosome targeting cationic amphiphilic drugs [30]. On the other hand, in gastric tumors with patchy HER2 membrane localization, the pharmacological depletion of caveolin 1 successfully forces HER2 to the cell surface for improved trastuzumab and pertuzumab efficacy [33,34]. The FLI-FRET method offers a new and unique way to look at and understand the biology of TZM–HER2 engagement at the multiscale, which may provide additional insights into the mechanisms of TZM action and resistance.

Here, we have extended the FLI-FRET assay to monitoring antibody–receptor-dependent target engagement in vitro and in vivo (Figure 5). Importantly, although the binding of donor-labeled probes or acceptor-labeled probes to their respective receptor occurs unrestrictedly, in a stochastic manner, FLI-FRET can only be detected when donor-labeled and acceptor-labeled probes are in close proximity within 2–10 nm distance. Thus, the occurrence of FRET may be due to the binding of donor-labeled and acceptor-labeled probes to the same homodimer receptor (intra-receptor FRET as shown in Figure 5) as well as to the binding of donor-labeled and acceptor-labeled probes to distinct receptor molecules clustered in close proximity (inter-receptor FRET). Therefore, this FLI-FRET TZM–HER2 drug–receptor binding assay is intrinsically limited to the quantification of only a fraction of ligand/receptor interactions, up to 50% [15,18,35]. However, the occurrence of receptor clustering may have the reverse effect, conceivably permitting the FRET signal to rise [16,19].

In this study, the goal was to test TZM–HER2 binding via FLIM-FRET imaging in vitro. In AU565 cells, donor TZM–AF700, in the absence of acceptor TZM–AF750, shows average lifetime values (≈1–1.1 ns), as shown previously for other NIR FRET probes [19,36], whereas in the presence of an acceptor, a significantly reduced lifetime is detected, indicating the occurrence of FRET due to TZM–HER2 interactions. Of importance, in AU565 cells, we found the shortest lifetime species at the plasma membrane, which may be due to the extensive HER2 clustering at the cell surface [37,38,39]. A wide range of lifetime values can also be detected in TZM-labeled endocytic structures likely indicating different states of TZM–HER2 interaction during binding at plasma membrane, internalization, and endocytic trafficking, recycling, and degradation. Nevertheless, even a single donor-labeled sample displayed significant lifetime heterogeneity behavior, which is consistent with the study showing a decrease of anti-HER2 affibody-DyLight750 lifetime upon binding to HER2-overexpressing tumors [40]. Currently, we are using phasor analysis to investigate the nature of TZM–AF700 lifetime heterogeneity [36]. One intriguing finding was the widespread heterogeneity of TZM–AF700 lifetime detected across HER2-expressing, e.g., AU565 cells, and non-HER2 expressing cells, such as MDA-MB-231 and MCF10A cells. MCF10A showed no uptake of TZM-AF700 or enough photons to allow for adequate FLIM fitting analysis, behaving as a clear negative FLIM-FRET control. In contrast, MDA-MB-231 cells displayed a non-specific TZM-AF700 uptake pathway, leading to internalization into intracellular structures that show a highly quenched lifetime donor signal in the presence or absence of an acceptor. These results suggest that TZM–AF700 may interact with an unknown target at the cell surface of MDA-MB-231 cells, leading to its subsequent internalization. In summary, FLIM imaging provides further information on the binding of fluorescently labeled probes to cancer cells that can be used to discriminate specific from non-specific interactions.

MFLI-FRET in vivo imaging proved that the quantification of TZM–HER2 engagement in AU565 tumor xenografts is feasible, offering a high fluorescence lifetime signal-to-noise ratio in the tumors and acceptable FRET levels in animals injected with A:D = 2:1 ratios, but not in that injected with donor only. Unexpectedly, we detected significant FRET levels in the livers and one bladder in animals injected with A:D = 2:1, indicating the occurrence TZM binding events. Since neither of these organs appear to overexpress HER2 on the plasma membrane, the likely explanation for the positive FRET level detected in the liver may be the occurrence of non-HER2-mediated TZM binding events. Indeed, a reduced FRET signal in donor only (A:D = 0:1) liver, a FRET-negative control, suggests that the A:D = 2:1 FRET signal detected in liver is due to TZM binding. Furthermore, several reports have shown the binding of TZM to mouse HER2 [41,42]. TZM-emtansine (T-DM1), an effective antibody–drug conjugate to treat advanced breast cancer, has also displayed significant liver toxicity [42]. Several PET imaging reports using radioactively labeled TZM have shown TZM liver binding above or at the threshold [43,44], suggesting that TZM may bind liver cells in mice and humans. Alternatively, lifetime reduction could be due to liver-specific microenvironments or low pH and proteinuria in urinary bladder, which is a normal condition in mice [45]. Moreover, variability in the signal detection can be attributed to animal positioning, physiology, or metabolism. However, the ability to detect quenched donor lifetime in non-HER2 expressing MDA-MB-231 cells in vitro suggests that some tissues/organs may contain non-HER2 specific targets that are recognizable by TZM–AF700, leading to lifetime reduction, which could confound FRET measurements in the absence of donor-only lifetime measurements, which is an important negative control for FLI-FRET experiments. More studies are necessary to determine the reason for the liver FRET TZM–AF700 signal, and we plan to explore it further in TZM–MFLI imaging whole-body tissue distribution experiments.

Here, we have expanded the MFLI-FRET methodology to monitor TZM binding to HER2, which is a highly clinically relevant breast cancer biomarker. Interestingly, TZM–AF700 was able to clearly delineate tumor margins due to a high rate of uptake, low background, and high signal-to-noise ratio of NIR MFLI-FRET in vivo imaging. In the future, we expect to use MFLI FRET to monitor anti-HER2 drug response longitudinally multiplexed with metabolic imaging. Such methodology will further the understanding of HER2-positive tumor physiology and its interplay with the microenvironment during targeted therapy. Moreover, we plan to perform FLI-FRET imaging using different combinations of anti-HER2 and anti-HER3 probes to quantify target engagement and modulation of HER2 homo- and HER2–HER3 heterodimerization in response to TZM treatment in vitro and in vivo. Therefore, MFLI-FRET is uniquely positioned to drive the development of the next generation of targeted drugs and to elucidate (multi-) drug resistance mechanisms by assessing simultaneously concentrations of active drugs at the tumor, discriminate between active and passive uptake, and monitor metabolic status.

## 4. Materials and Methods

### 4.1. Cell Culture

All cell lines used in this study were obtained from ATCC (Manassas, VA, USA). HER2-overexpressing breast cancer cell line AU565 was cultured in RPMI medium supplemented with 10% fetal bovine serum (ATCC), 10 mM HEPES and 50 Units/mL/50 μg/mL penicillin/streptomycin (Thermo Fisher Scientific, Waltham, MA, USA). Triple negative MDA-MB-231 cells were cultured in Dulbecco’s modified Eagle’s medium (Thermo Fisher Scientific, Waltham, MA, USA) supplemented with 10% fetal calf serum (ATCC), 4 mM L-glutamine (Thermo Fisher Scientific), 10 mM HEPES (Sigma, St. Louis, MO, USA), and penicillin/streptomycin (50 Units/mL/50 µg/mL, Thermo Fisher Scientific) at 37 °C and 5% CO_2_. The cells were used within passage ten to prevent phenotype drift. Mammary epithelial cells MCF10A were cultured in DMEM/F12 with 5% horse serum, 20 ng/mL EGF, 0.5 mg/mL hydrocortisone, 100 ng/mL cholera toxin, 10 µg/mL bovine insulin, and penicillin/streptomycin.

### 4.2. TZM Labeling 

Humanized monoclonal antibody against HER2 trastuzumab was obtained from Genentech and conjugated to AF700 or AF750 (Thermo Fisher Scientific) through monoreactive *N*-hydroxysuccinimide ester to lysine residues in the presence of 100 mM Na bicarbonate, pH 8.3, according to manufacturer’s instructions. The probes were purified by using Amicon Ultra-4 centrifugal filter units (Sigma Z648035, MWCO 30 kDa). After extensive washes with PBS, the probes are reconstituted in PBS, protein concentration is measured and normalized to 1 mg/mL. The degree of labeling of the probes was assessed by spectrophotometer DU 640 (Beckman Coulter, Fullerton, CA, USA). The average degree of labeling was no more than 2 fluorophores per molecule. All probes were filter sterilized and stored at 4 °C without preservative.

### 4.3. Uptake and Immunofluorescence Assays Using Confocal Microscopy

For uptake experiments, cells were plated on 8 well µ-slides (Ibidi, Fitchburg, WI, USA) at 50,000 cells per well and cultured overnight followed by 24 h incubation with TZM-AF700 and Tf-AF568 (Thermo Fisher) in cell culture medium. Cells were fixed for 15 min with 4% paraformaldehyde (PFA) and processed for immunocytochemistry using rabbit monoclonal HER2 antibody (Cell Signaling cat#2165, 1:500), rat HER2-AF488 (Bio Rad MCA1788A488 1:50), rabbit monoclonal EEA1 antibody (Cell Signaling cat#3288, 1:500), mouse monoclonal EEA1 (BD Biosciences cat#610457 1:500), mouse monoclonal TfR antibody (Abcam ab38171, 1:500), or rabbit polyclonal TfR antibody (Abcam ab84036, 1:500). Secondary antibodies F(ab) goat anti-mouse AF647, AF568, goat anti rabbit AF488, AF555, AF647 (Thermo Fisher Scientific) were used at 1:500 dilution. Samples were imaged on Zeiss LSM 880 confocal microscope using identical settings across all samples.

### 4.4. NIR FLIM-FRET Microscopy

Cells were plated on MatTek 35 mm glass-bottom plates and cultured overnight. Cells were incubated with TZM–FRET pair probes in culture medium for 24 h at 37 °C: TZM–AF700 (20 µg/mL), TZM–AF750 (40 ug/mL), using an Acceptor/Donor ratio 0:1, 0.5:1, 1:1, 2:1, and 3:1. After TZM internalization, cells were washed with HBSS buffer, fixed with 4% PFA, and stored in DHB solution for imaging (phenol red-free DMEM, 5 mg/mL bovine serum albumin, 4 mM L-glutamine, 20 mM HEPES, pH 7.4). NIR FLIM-FRET microscopy was performed on a Zeiss LSM 880 Airyscan NLO multiphoton confocal microscope using an HPM-100-40 high speed hybrid FLIM detector (GaAs 300–730 nm; Becker & Hickl) and a Titanium/Sapphire laser (Ti:Sa) (680–1040 nm; Chameleon Ultra II, Coherent, Inc., Santa Clara, CA, USA) as described in [20]. A Semrock FF01-716/40 band pass filter and a Semrock FF01-715/LP blocking edge long-pass filter were inserted in the beamsplitter assembly to detect the emission from AF700 and to block scattered light, respectively. The 80/20 beamsplitter in the internal beamsplitter wheel in the LSM 880 was used to direct the 690 nm excitation light to the sample and to pass the emission fluorescence to the FLIM detector. The data were analyzed by two-component exponential fitting using SPCImage software (Becker & Hickl GmbH, Berlin, Germany). A χ2 fitness test was used to determine the validity of the fit, providing χ2 values <1.5 for all pixels. Ten pixels from at least three different images per ratio were used for analysis. The data are presented as average and standard deviation.

### 4.5. Animal Experiments

All animal procedures were conducted with the approval of the Institutional Animal Care and Use Committee at both Albany Medical College and Rensselaer Polytechnic Institute. Animal facilities of both institutions have been accredited by the American Association for Accreditation for Laboratory Animals Care International. Tumor xenografts were generated by injecting 10 × 10^6^ AU565 cells in phosphate-buffered saline (PBS) mixed 1:1 with Cultrex BME (R&D Systems Inc, Minneapolis, MN, USA) into the right inguinal mammary fat pad of female 5-week-old athymic nude mice (CrTac:NCr-Foxn1^nu^, Taconic Biosciences, Rensselaer, NY, USA). The subcutaneous tumors were allowed to grow for 4–5 weeks and were monitored daily. TZM-AF700 and TZM-AF750 (20 and 40 µg, respectively) were injected retro-orbitally in anesthetized mice.

### 4.6. Wide-Field Macroscopic Fluorescence Lifetime Imaging Platform

MFLI was performed on a wide-field time-domain fluorescence lifetime imaging tomographic system, as described previously [46]. Briefly, the system excitation source was a tunable Ti-Sapphire laser (Mai Tai HP, Spectra-Physics, CA, USA). The spectral range was 690–1040 nm with 100-fs pulses at 80 MHz. The laser was coupled to a digital micro-mirror (DMD) device (DLi 4110, Texas Instruments, TX, USA), which produced a wide-field illumination over an 8 × 6 cm area with 256 grayscale levels encoding at each pixel. The wide-field illumination was spatially modulated by controlling the DMD via Microsoft PowerPoint to ensure optimal signal detection over the whole animal body [47]. The detection system was a time-gated intensified CCD (ICCD) camera (Picostar HR, Lavision GmbH, Bielefeld, Germany). The gate width on the ICCD camera was set to 300 ps. The Instrument Response Function (IRF) and fluorescence signals were collected with a 40 ps time interval over a 7.0 ns time window. The total number of gates acquired was 175, and the maximum range of detection was 4096 photons per pixel per gate. The multichannel plate gain (MCP) employed for signal amplification was set to 550 V for the whole imaging session. In this study, imaging was performed in reflectance mode. The laser excitation for AF700 was set at 705 nm and the emission filters were 720 ± 6.5 nm (FF01-720/13-25, Semrock, IL, Rochester, NY, USA) and 715 nm long pass filter (Semrock, FF01-715/LP-25). The IRF was measured by using a full field pattern projected on diffuse white paper and acquiring the temporal point spread function (TPSF) without using an emission filter. The imaging platform was equipped with an isoflurane anesthesia machine and a digitally controlled warming pad.

### 4.7. Bi-Exponential Fitting to Extract FRET Donor Fraction

The FRET donor fraction indicating fraction of population of AF700–TZM (donor) in close proximity of AF750–TZM (acceptor), allowing FRET to occur (FRET range, 2–10 nm) within a region of interest (ROI), was quantified by fitting the fluorescence decays in each pixel of ROI to the bi-exponential model:

I(t) = IRF(t) ⊗ (A_1_ e^(−t/τ_1_) + A_2_ e^(−t/τ^2^)). (1)

Here, I(t) represents the fluorescence decay, IRF(t) is the instrument response function inherent to the system and collected on a piece of diffuse white paper, A_1_ and A_2_ are the FRET and non-FRET donor fractions respectively, τ_1_ and τ_2_ are the quenched and unquenched donor lifetimes, t is time, and **⊗** represents convolution. The tail portion of the decays (99–2% of the peak value) in each pixel of an ROI is fit to the bi-exponential model to extract the FRET donor fraction (A_1_) using the Matlab function fmincon for least squares minimization of the cost function. The fluorescence decays from the MFLI system were analyzed with the same fitting parameters and smoothing with Anscombe filtering. The average values and standard deviations are reported.

### 4.8. Immunohistochemistrycoherent,Inc

Excised tumors were fixed in formalin, paraffin embedded, and processed for IHC. Epitope retrieval was performed by boiling deparaffinized and rehydrated 5µm sections in 1 mM EDTA pH 8.0 for 30 min. IHC staining was carried out using a standard protocol from Vectastain ABC Elite kit (Vector Labs, Burlingame, CA cat#PK-6101). Vector NovaRED (Vector Labs) was used as a peroxidase substrate. Tissue sections were counterstained with Methyl Green (Sigma, cat# M8884). Hematoxylin Eosin stain was used for basic histology. Primary antibodies were as followed: rabbit monoclonal HER2 1:800 (Cell Signaling, #2165), rabbit monoclonal HER3 1:250 (Cell Signaling, #12708), rabbit monoclonal TZM 1:100 (R&D Systems, MAB95471-100). Brightfield images were acquired using Olympus BX40 microscope equipped with Infinity 3 camera (Lumenera Inc., Ottawa, ON, Canada).

### 4.9. Statistical Analysis

The statistical significance of the data was tested with unpaired Student’s t tests. Differences were considered significant if the p-value was less than 0.05. Error bars indicate standard deviation or 95% confidence interval.

## Supplementary Materials

The following are available online, Figure S1: Colocalization analysis of HER2 and TZM with endocytic markers, Figure S2: HER2 expression and colocalization with TZM in AU565 vs MDA-MB-231 cancer cells, Figure S3: Comparison of NIR TZM-HER2 and Tf-TfR FLIM FRET in AU565 cells, Figure 4S: Representative TCSPC images of fluorescence intensity and mean lifetime map (τ_m_) in MCF10A cells treated with TZM-AF700 and TZM-AF700/750.
