# Supplementary material for: Quantification of Trastuzumab–HER2 Engagement In Vitro and In Vivo
Source: Molecules. 2020 Dec 17;25(24):5976. doi: 10.3390/molecules25245976 (PMC7767145; doi:10.3390/molecules25245976)
Supplement: Supplementary file 1 [file molecules-25-05976-s001.pdf]

molecules

MDPI

Article

Quantification of Trastuzumab–HER2 Engagement In Vitro and In Vivo

Alena Rudkouskaya [1], Jason T. Smith [2], Xavier Intes [2] and Margarida Barroso [1,*]

[1] Department of Molecular and Cellular Physiology, Albany Medical College, Albany, NY 12208, USA; rudkoua@amc.edu
[2] Center for Modeling, Simulation, and Imaging in Medicine, Rensselaer Polytechnic Institute, Troy, NY 12180, USA; smithj28@rpi.edu (J.T.S.); intesx@rpi.edu (X.I.)
* Correspondence: barrosm@amc.edu; Tel.: +1-518-573-3816

Supplemental Material

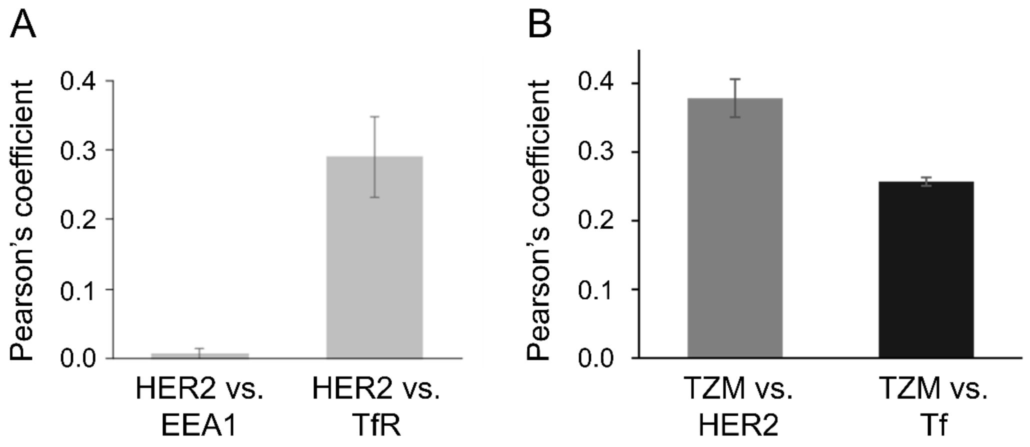

Figure S1. Colocalization analysis of HER2 and TZM with endocytic markers. (A) AU565 cells were subjected to immunostaining with anti-HER2, anti-EEA1 and anti-TfR as described in Figure 1A. Pearson coefficient was calculated on images shown in Figure 1A. (B) AU565 cells were subjected to 24 h uptake with 5 μg/mL TZM-AF700 and 5 μg/mL Tf-AF568 and processed for immunostaining for HER2 as shown in Figure 1B. Pearson coefficient was calculated on images shown in Figure 1B. Collected z-stacks were analyzed for colocalization in Imaris (Bitplane) software, as described in [26]. Data presented as mean and standard deviation, n=3-5.

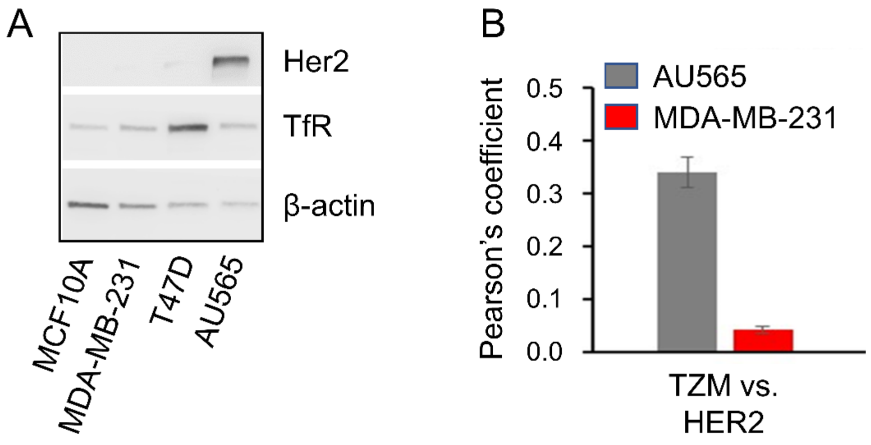

Figure S2. (A) Representative Western blot of HER2 and TfR expression in breast cancer cells and non-cancerous MCF10A cells. (B) AU565 and MDA-MB-231 cells were subjected to 24 h uptake with 20 µg/mL TZM-AF700 and processed for immunostaining for HER2. Pearson coefficient was calculated on images shown in Figure 2B. Collected z-stacks were analyzed for colocalization in Imaris (Bitplane) software, as described in [26]. Data presented as mean and standard deviation, n=5.

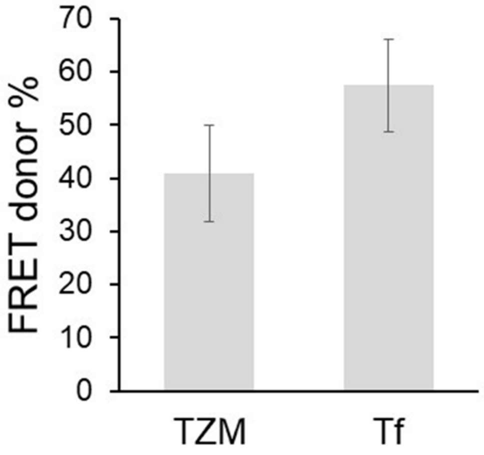

Figure S3. Comparison of NIR TZM-HER2 and Tf-TfR FLIM FRET in AU565 cells. Data presented as mean and standard deviation, n=10 points from five separate ROIs.

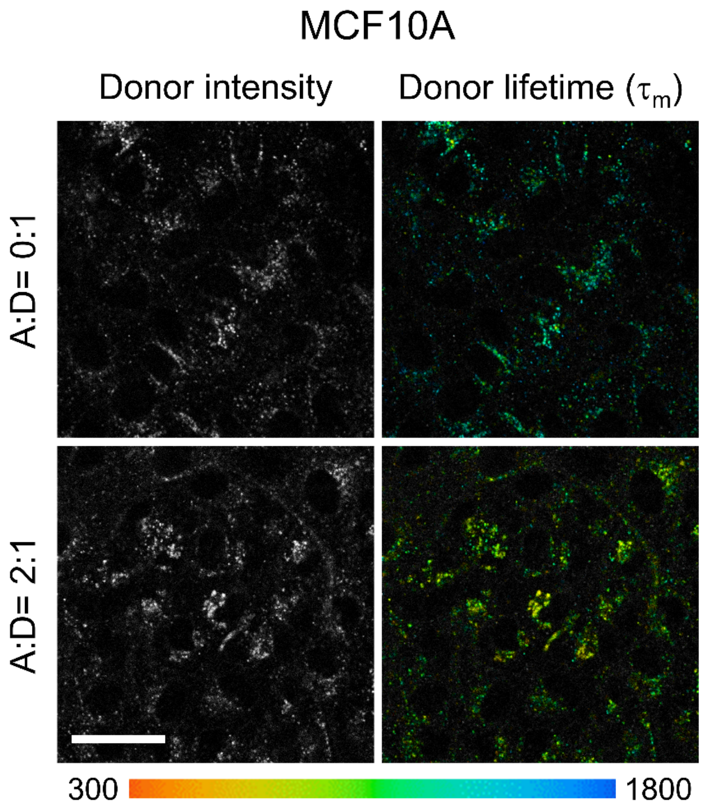

Figure S4. Representative TCSPC images of fluorescence intensity and mean lifetime map (τ_m) in MCF10A cells treated with TZM-AF700 (Acceptor: Donor =0:1), TZM-AF700 and TZM-AF750 (A:D=2:1) pseudo-color range= 500-1,800 ps. Photon counts were insufficient for adequate fitting analysis.
